# Influence of Fatigue on the Rapid Hamstring/Quadriceps Force Capacity in Soccer Players

**DOI:** 10.3389/fphys.2021.627674

**Published:** 2021-02-05

**Authors:** Qingshan Zhang, Baptiste Morel, Robin Trama, Christophe A. Hautier

**Affiliations:** ^1^Laboratoire Interuniversitaire de Biologie de la Motricité, Université Claude Bernard Lyon 1, Villeurbanne, France; ^2^Laboratoire Interuniversitaire de Biologie de la Motricité, Université Savoie Mont Blanc, Chambéry, France

**Keywords:** peak torque, fatigue, rate of torque development, rate of EMG rise, H/Q ratio

## Abstract

The objective of this study was to examine the effect of fatigue on maximal and rapid force capacities and muscular activation of the knee extensors and flexors. Seventeen professional soccer players volunteered to participate in this study. Peak torque (T_peak_) and rate of torque development (RTD) of knee flexor (90°. s^–1^, −30°. s^–1^) and extensor (90°. s^–1^) muscles were measured before and after fatigue (i.e., 30 maximal knee extension and flexion repetitions at 180°s^–1^) performed on an isokinetic dynamometer. Hamstring to quadriceps peak strength and RTD ratios were calculated. Besides, using surface EMG, the mean level of activation (RMS_mean_), Rate of EMG Rise (RER), and EMG Frequency-Time maps were measured on quadriceps and hamstring muscles. Following fatigue, T_peak_, RTD, RER declined significantly in the two muscle groups (all *p* < 0.05) without modification of RMS_mean_. No decrease in conventional and functional H/Q ratios was observed after fatigue except for a significant increase in the *H*_ecc30_/*Q*_con180_ ratios (1.03 ± 0.19 vs. 1.36 ± 0.33, *p* < 0.001). Besides, the RTD H/Q ratios decreased significantly after fatigue, and the statistical parametric mapping analysis (SPM) performed on the EMG/angle curves, and EMG Frequency-Time maps showed that fatigue strongly influenced the muscle activation during the first 100 ms of the movement, following the higher EMG frequency component shift toward the lower frequency component. Our results show that the reduction of RTD and RER during the first 100 ms of the contraction after fatigue exercise makes more sense than any H/Q ratio modification in understanding injury risk in soccer players.

## Introduction

Hamstring strain injuries (HSIs) and anterior cruciate ligament (ACL) rupture are, respectively, the most prevalent non-contact injuries (Ekstrand et al., 2011a) and the most serious injuries considering time to return to play (Walden et al., 2016) in professional soccer players (Ekstrand et al., 2011b). During tasks such as sprinting, kicking or cutting, co-contraction of the hamstring and quadriceps muscles increases knee joint stability and prevents excessive knee joint constraints. That is why any deficit in hamstring strength or activation pattern may limit the protective effect of co-contraction on ACL and hamstring muscles. Thus, HSIs and ACL ruptures are frequently associated with strength imbalance between knee flexor and extensor muscles (Croisier et al., 2008; Alhammoud et al., 2019; Baroni et al., 2020). For this reason, isokinetic dynamometry is currently used by most of the professional soccer teams to calculate conventional and functional H/Q ratios to predict the strength imbalance between knee flexor and extensor muscles. Nevertheless, the predictive value of H/Q torque ratios for ACL ruptures and HSIs remains poor, maybe because they do not mimic the physiological and biomechanical ecological constraints such as joint angular position, contraction time, or fatigue (Croce and Miller, 2006; Coratella et al., 2015; Evangelidis et al., 2015; De Ste Croix et al., 2017; Alhammoud et al., 2019).

Accordingly, it has been suggested that modeling H/Q ratios with angle-specific metrics through range of motion may provide a more specific tool for analyzing thigh muscle balance. A recent study demonstrated that statistical parametric mapping analyses identified angle-specific differences between female and male elite skiers that could not be evidenced when analyzing only maximal torques and reconstructed ratios (Alhammoud et al., 2019). Thus, knee angle and muscle length are essential factors that have to be taken into account when interpreting the H/Q ratios. Moreover, considering that fatigue increases the injury risk in soccer, the modifications of this dynamic H/Q ratio under fatigue are of great importance.

Recent studies have suggested that the rate of torque development (RTD) during the first milliseconds of the contraction represents another functional outcome related to performance and injury risk (Alhammoud et al., 2018; Ishoi et al., 2019). For example, the study by Ishoi et al. (2019) indicates that early phase (0–100 ms) rapid force capacity of the hamstring muscles strongly influenced the acceleration capacity of elite soccer players (Ishoi et al., 2019). Besides, RTD is considered to be very important in the ability to stabilize the musculoskeletal system in response to mechanical perturbation (Angelozzi et al., 2012; Alhammoud et al., 2018). As there is no systematic link between the maximum force capacity and the relative RTD, screening for the risk of injury by measuring the traditional H/Q ratio can be difficult because of this inability to measure the capacity of rapid stabilization of the knee which may influence the risk of injury (i.e., HSIs, ACL injury). Furthermore, neuromuscular fatigue has been identified as a potential risk factor of HSIs (Croisier et al., 2008), considering that HSIs preferentially occur in the later stage of the match (Hawkins et al., 2001; Small et al., 2009). Fatigue can be defined as a reduction in the force-generating capacity of the neuromuscular system, regardless of the level of force required (Morel and Hautier, 2017). Match-related fatigue in professional soccer players induces a reduction of jump ability, sprint performance (Krustrup et al., 2006), peak torque in knee extension and flexion (Small et al., 2010; Marshall et al., 2014) and RTD (Marshall et al., 2014; Grazioli et al., 2019). As hamstring strength is strongly affected after a simulated and a real soccer match, it may contribute to the strength imbalance between hamstring and quadriceps (Cohen et al., 2013; Grazioli et al., 2019), and may be considered as an injury risk factor (Lee et al., 2018). That way, Small et al. (2009) revealed a significant reduction in combined maximal hip flexion and knee extension angle resulting in shorter hamstring length during sprint running after soccer-specific fatigue, which potentially increase predisposition to hamstring strain (Small et al., 2009). It is also worth noting that a previous study reported a greater torque loss in hamstring compared to quadriceps muscles during 50 flexions-extensions at 180°.s^–1^ in concentric mode inducing H/Q ratio deterioration (Sangnier and Tourny-Chollet, 2007). In addition, RTD has been demonstrated to be highly sensitive to fatigue (Morel et al., 2015; Grazioli et al., 2019). For instance, Grazioli et al. (2019) reported that hamstring explosive force is more affected than maximal force after a professional soccer match (Grazioli et al., 2019). Thus, the RTD H/Q ratio has been demonstrated to be an interesting way to characterize muscle function around the knee (Zebis et al., 2011; Greco et al., 2013). For this reason, studying angle-specific H/Q ratio and RTD H/Q ratio modifications after fatigue in soccer players appears to be a promising way to better understand and prevent HSIs and ACL injury occurrence.

Hence, the purpose of this study was to assess the effect of fatigue on angle-specific H/Q ratio and RTD H/Q ratio in soccer players. Furthermore, the present study will analyze the changes in muscle activation between the hamstring (i.e., Semitendinosus and Biceps femoris) and quadriceps (i.e., Rectus femoris and Vastus lateralis and Vastus medialis) under fatigue. We hypothesized that (i) the angle-specific H/Q ratio would be altered in certain knee angles without modification of the traditional H/Q ratio (i.e., conventional and functional H/Q ratio) and (ii) diminution of RTD (i.e., knee extensor and flexor) and RTD H/Q ratio would be associated with muscular deficit activation during the initial phase of contraction (i.e., 50, 100 ms).

## Materials and Methods

### Participants

Seventeen French semi-professional soccer players (Height: 173.5 ± 4.1 cm, Mass: 73.3 ± 6.3 kg, Age: 23.9 ± 2.2 years, training volume: 12 ± 2.2 h. wk^–1^) playing at a national level volunteered to participate in this study. Included subjects had no history of traumatic lower limb injuries (e.g., ACL rupture, hamstring injuries) for at least 1 year before the study. The cumulated training and match time per season attained 330 h (85% training and 15% competing). Leading up to the experiments, participants followed their regular training program avoiding any strenuous and high charge or match 48 h before the study. The sample size (*n* = 17) was estimated on the basis of the results of Sangnier and Tourny-Chollet (2008), accepting a confidence rate of 95% and a margin of error of 5% with a statistical power of 0.90, and looking for a medium effect size of 0.5 (Sangnier and Tourny-Chollet, 2008).

### Experimental Design

**FIGURE 1:**
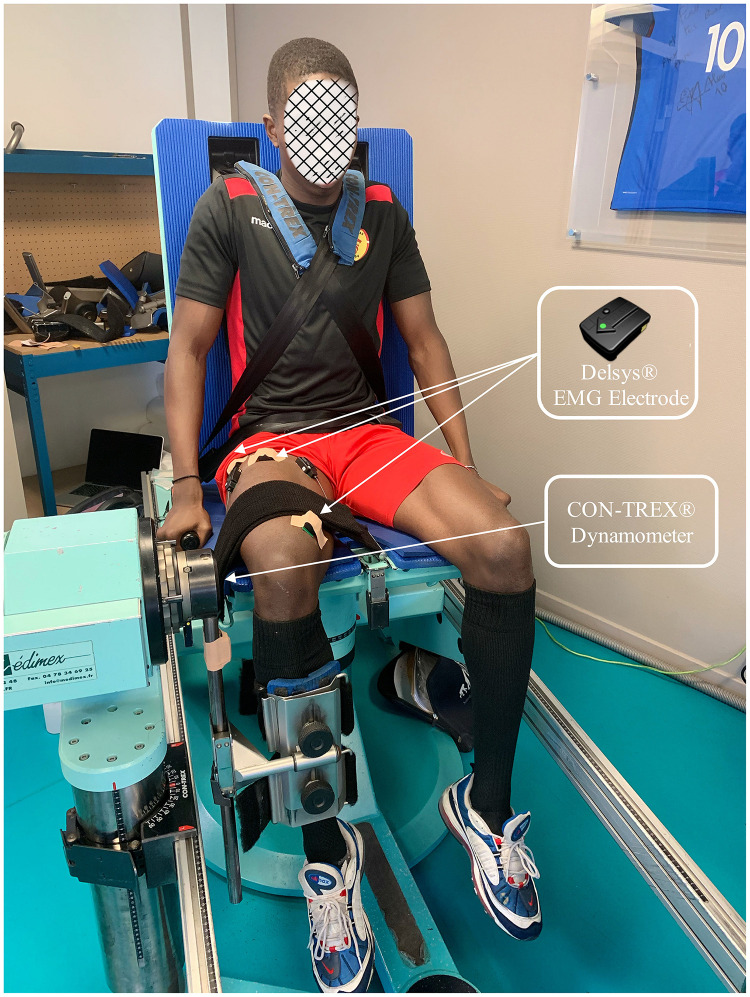
An example of participant experimenting.

A randomized repeated measurement pre-/post-fatigue study design was used for this research (Figure 1). Isokinetic measures were performed on the dominant lower limb, which was determined by the preferred leg in kicking. The participants performed a general warm-up composed of 10 min cycling (1 watt.kg^–1^, 70–80 RPM) and mobility movements. After that, sEMG electrodes (Delsys Trigno^TM^ Wireless EMG System, bipolar Ag: AgCI surface, 2 cm inter-electrode distance) (Delsys, Inc., Boston, United States) were fixed on the rectus femoris (RF), vastus lateralis (VL), vastus medialis (VM), biceps femoris long-head (BF), and semitendinosus (ST) muscles following the SENIAM guidelines. The skin was shaved and cleaned using an alcohol wipe before affixing the sEMG electrode. The sampling rate was 2000 Hz, and the standard mode of rejection ratio was greater than 80 dB.

Once the participants were equipped with the EMG electrodes, they were afterward seated on the Contrex isokinetic dynamometer (Contrex, CMV AG, Dübendorf, Switzerland) with flexed hips at 80° (0° = full hip extension), and standard stabilization strapping was placed across the chest, pelvis, and distal thigh. The sampling frequency was 256 Hz. The axis of the dynamometer was visually aligned with the lateral femoral condyle. The range of movement was set from 100° of knee flexion (starting position) to 20° (0° was determined as the anatomical maximal voluntary knee extension for each participant). The subjects were required to grasp the handles of the dynamometer chair (Figure 1).

### Experimental Sessions

Before the isokinetic testing, all participants were asked to perform a familiarization session. During this session, the participants were asked to complete eight continuous reciprocal knee extensions and flexions between 50 and 60% of their maximal at two velocities 90°.s^–1^, and 180°.s^–1^ followed by eight hamstring eccentric contractions at −30°.s^–1^. This allowed the participants to adapt to the isokinetic contractions and determined the quality of the sEMG signal. Before assessments, the gravity compensation procedure was performed according to the manufacturer’s instructions. After ∼3 min of passive recovery (Parcell et al., 2002), the participants performed the pre-fatigue tests, including five maximal knee extensors and flexors concentric contractions at 90°.s^–1^ and five maximal knee flexors eccentric contractions at −30°.s^–1^. Two minutes of recovery were included between concentric and eccentric sets. After 2 min of passive recovery, subjects performed three maximal knee extensors and flexors concentric contractions at 180°.s^–1^ immediately followed by the fatiguing exercise composed of 24 contractions at the same velocity. Immediately after the fatigue session, subjects repeated the pre-fatigue tests but with no rest between the contractions. During the entire protocol, the participants were verbally encouraged to give their maximal effort by the same manipulator.

### Measurements and Data Analysis

Raw torque-angle curves were extracted from the original instantaneous torque dataset using custom routines to resynchronize the signal per degree of knee angle. Raw data were processed in MATLAB (MathWorks, version 2018b, Natick, MA, United States), a 2nd low-pass 20 Hz was used to filter. Furthermore, a 1D polynomial spline fitting method was used to interpolate torque and angle values for each contraction to guarantee the same data length. The three best trials were extracted for pre- and post-fatigue tests, and the first and last concentric contractions of the fatigue protocol were taken into account. The threshold of torque was fixed at 1% of local peak torque for determining the onset:offset of muscle contraction.

### Non-angle Specific Peak Torque (T_peak_) and H/Q Ratios

Peak torque (T_peak_) at 90°.s^–1^ 180°.s^–1^ and −30°.s^–1^ were calculated as the average value of three contractions (the best ones of the five contractions for 90°.sec-1 and −30°.sec-1) performed before and after the fatigue protocol both for knee extensors (KE), and knee flexors (KF). Conventional non-angle specific H/Q ratios were calculated by dividing knee flexion concentric peak torque by knee extension concentric peak torque at 90°.s^–1^ (H/Q_con90_) and 180°.s^–1^ (H/Q_con180_). A functional non-angle specific H/Q ratio was calculated by dividing knee flexion eccentric Peak torque at −30°.s^–1^ by knee extension concentric peak torque at 90°.s^–1^ (H/Q_func90_) and 180°.s^–1^ (H/Q_func180_).

### Angle-Specific Peak Torque and Angle-Specific H/Q Ratios

All angle-specific measures were represented only between 30 and 90° to account for the acceleration and deceleration phases and keep within the iso-velocity period (Alhammoud et al., 2019). Based on these torque-angle curves, angle-specific conventional and functional H/Q dynamic ratios were continuously calculated by dividing hamstring torque by quadriceps torque at the same knee angle across the analyzed range of motion. Two conventional angle-specific dynamic ratios (H/Q_conv90θ_ at 90°.s^–1^ and H/Q_conv180θ_ at 180°.s^–1^) and two functional angle-specific dynamic ratios were calculated for knee extension (hamstring torque at −30°.s^–1^ divided by quadriceps torque at 90°.s^–1^; H/Q_func90θ_ and hamstring torque at −30°.s^–1^ divided by quadriceps torque at 180°.s^–1^; H/Q_func180θ_). A 1D polynomial spline fitting method was used to interpolate torque and angle values for all the curve between 30 and 90° to normalize the torque: angle curves on the same number of points.

### Rate of Torque Development and RTD H/Q Ratios

The absolute RTD (Nm.s^–1^) was calculated from linear slope of the torque-time curve (i.e., Δtorque/Δtime) (Aagaard et al., 2002) at the 0–50 ms and 0–100 ms time interval during dynamic isokinetic concentric contractions at 90°.s^–1^, 180°.s^–1^ for the knee extensor and flexor muscles and during isokinetic eccentric contractions at −30°.s^–1^ for the knee flexors (Alhammoud et al., 2018). Besides, the RTD H/Q ratios were calculated by dividing the RTD of knee flexor by the RTD of knee extensor corresponding time intervals both for the 50 ms (RTD_50 ms_ H/Q Ratio) and 100 ms (RTD_100 ms_ H/Q Ratio).

### EMG Variables

The EMG Signal Processing of five muscles (e.g., BF, ST, RF, VL, VM) was performed using MATLAB software (R2018b, The MathWorks Inc., Natick, MA, United States). The root mean square values (RMS) were calculated with a 150-ms moving rectangular window based on the rectified EMG to determine the signal amplitude. The onset and offset of the EMG bursts were determined using a threshold of 5% of the muscle peak maximal activation found within the analysis period. RMS EMG signals were normalized to the peak RMS value measured for each muscle during the pre-fatigue maximal contraction at 90°.s^–1^. A mean RMS value (RMS_mean_) was then calculated between onset and offset for the five muscles during pre-and post-fatigue tests. The calculation of the rate of EMG rise (RER) was realized on a smoothed RMS curve (6 Hz low-pass fourth-order Butterworth filter) and calculated by the slope of the RMS-Time envelope for 0–50 ms and 0–100 ms time intervals relative to the activation onset for the five muscles during pre-and post-fatigue tests (Morel et al., 2015).

A continuous wavelet transform (CWT) was made on raw EMG signals to investigate time-frequency domain for each muscle before and after fatigue, using a Morse mother wavelet with γ = 3 and *P*^2^ = 60 (Lilly and Olhede, 2009). The frequencies ranging from 20 to 500 Hz were analyzed. The pseudo frequency of each scale was obtained using the center of the frequency of each wavelet, and as the frequencies were logarithmically spaced, the maps were linearly interpolated by 4 Hz in the frequency domain. The time was normalized for all the maps corresponded to 200-time points. Thus, the number of nodes of the maps corresponded to 200 [time] × 94 [frequency] = 18800 nodes. The modulus of the coefficient given by the wavelet transform was computed for each node. The frequency that split the spectrum in half was defined as the median frequency.

### Statistical Analysis

Before performing the statistical analysis, the Shapiro–Wilk test and Levene’s test were respectively used to assess the normality of data and the equality of variance for isokinetic data and EMG data. Thereafter, a two-way (Angular velocity^∗^Muscle) repeated measures (Pre- and Post-fatigue test) analysis of variance ANOVA was used on T_peak_, RTD_100 ms_, RMS_mean_, RER_100 ms_. Holm’s Bonferroni *post hoc* test with correction of the *p*-value was used for multiple comparisons to determine the interaction effect between the test (Pre-fatigue vs. Post-fatigue condition), muscle (knee flexor vs. extensor for isokinetic variable and five-goal muscle (e.g., BF, ST, RF, VL, and VM) for EMG data and angular velocity (90°.s^–1^, 180°.s^–1^). Besides, Student’s paired *t*-test was applied to analyze the different H/Q ratios and eccentric peak torque (−30°.s^–1^) before and after fatigue. Statistical significance was set at *p* < 0.05. All statistical procedures were performed with IBM SPSS 26 (IBM Corporation, Armonk, NY, United States). All the data are presented as mean ± SD, with a 95% confidence interval (95% CI).

Using SPM–1D package (^©^Todd Pataky, version M 0.1) in MATLAB (The MathWorks Inc, R2018b, Natick, MA, United States) was conducted to performs Statistical Parametric (Pataky, 2010). One dimentional paired *t*-test SPM statistics were performed to determine the main effects of fatigue on the angle-specific torque, angle-specific H/Q ratio, smoothed RMS curve, and a *p*-value was calculated for clusters crossing the critical threshold, with significance set at *p* < 0.05 (Pataky et al., 2013). Besides, SPM analysis was conducted in two dimensions on the EMG Frequency-Time maps obtained from the wavelet transform. The mean maps of each subject’s three contractions were considered for the analysis. As a 1D continuum is required to perform the SPM analysis, the maps were flattened into a 1D continuum for the analysis and then reshaped to their original dimensions for interpretation. For concentric contractions, maps were analyzed with a two-way ANOVA with two repeated measures (Fatigue: Pre vs. Post; Velocity: 90°.s^–1^ vs. 180°.s^–1^) and *post hoc* tests as paired *t*-tests with Bonferroni correction. An alpha error of 0.01 (0.05/5) was set as five muscles were analyzed. For eccentric contractions, paired *t*-tests were performed to compare maps (Pre vs. Post) with an alpha error at 0.025 as two muscles were considered. To define the threshold of the statistical tests performed, permutation tests were used with 10000 permutations (Nichols and Holmes, 2002).

## Results

### Peak Torque (T_peak_) and H/Q Ratios

**FIGURE 2:**
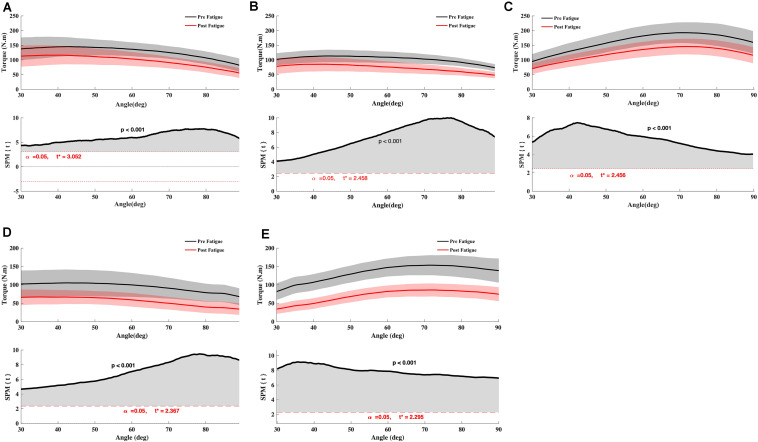
Effect of fatigue on knee flexor and extensor torque. **(A)** Knee Flexor at −30°.s^–1^, **(B)** Knee Flexor at 90°.s^–1^, **(C)** Knee Extensor at 90°.s^–1^, **(D)** Knee Flexor at 180°.s^–1^, **(E)** Knee Extensor at 180°.s^–1^. Paired *t* SPM analysis. Mean ± 95% confidence interval angle-series (upper panel) and corresponding SPM vector field statistical analysis (lower panel). The red dotted line represents the critical random field, the threshold value, *t* statistical set for α = 0.05. The *p* < 0.05 portion of the curve is shaded in gray.

The knee extension T_peak_ reduced significantly after fatigue at 90°.s^–1^ and 180°.s^–1^ (all *p* < 0.05) (Table 1). The knee flexion T_peak_ reduced significantly after fatigue at 90°.s^–1^, 180°.s^–1^, and −30°.s^–1^ (all *P* < 0.05) (Table 1). In addition, there was a significant interaction effect for different velocity (all *p* < 0.05). Holm’s Bonferroni *post hoc* tests indicated that the reduction of force was higher at an angular velocity of 180°.s^–1^ than 90°.s^–1^ (all *p* < 0.01). Besides, the *t*-test SPM analysis on torque-angle curves indicated a significant decrease in torque for knee extensor and flexor irrespective of knee angle (Figure 2). Moreover, the conventional ratios at 90°.s^–1^ and 180°.s^–1^ and the functional *H*_ecc30_/*Q*_conc90_ ratio were not affected by the fatigue protocol (*p* > 0.05) (Table 2). In contrast, the functional *H*_ecc30_/*Q*_con180_ ratio increased after fatigue (*p* < 0.001) (Table 2).

**TABLE 1:** Effect of fatigue on peak torque (T_peak_) in Nm, rate of torque development (RTD) in Nm. s^–1^.

Velocity [°. s^–1^]	Muscle	Variable	Pre-fatigue	Post-fatigue	Mean Difference [95% CI]	Change [%]
	Knee Extensor	T_peak_	153.7 ± 29.6	92.1 ± 11.2	−61.6 [47.1 − 76.2]	−40.1%***
180		RTD_50 ms_	847.6 ± 348.4	420.7 ± 176.1	−427.8 [221.4 – 632.3]	−50.5%***
		RTD_100 ms_	1002.8 ± 341.0	509.8 ± 173.2	−493.0 [284.3 – 701.5]	−49.2%***
	Knee Flexor	T_peak_	112.8 ± 30.2	69.1 ± 16.5	−43.7 [27.1 − 60.3]	−38.7%***
		RTD_50 ms_	744.1 ± 316.7	387.1 ± 142.1	−357.0 [151.6 – 562.4]	−47.9%**
		RTD_100 ms_	799.2 ± 294.4	465.2 ± 191.1	−334.1 [125.4 – 542.7]	−41.8%**
90	Knee Extensor	T_peak_	201.1 ± 36.7	150.6 ± 25.7	−50.5 [31.9 − 69.1]	−25.1%***
		RTD_50 ms_	1131.1 ± 457.8	734.4 ± 419.4	−396.6 [191.2 – 602.0]	−35.1%***
		RTD_100 ms_	1174.4 ± 365.8	795.1 ± 386.2	−379.4 [170.7 – 587.9]	−32.3%***
	Knee Flexor	T_peak_	126.8 ± 24.7	92.3 ± 22.9	−34.5 [16.5 − 52.5]	−27.2%**
		RTD_50 ms_	791.1 ± 318.7	554.7 ± 325.9	−236.5 [31.1 – 441.8]	−29.9%*
		RTD_100 ms_	924.7 ± 339.8	738.3 ± 337.2	−186.4 [−22.3 – 395.0]	−20.2%*
−30	Knee Flexor	T_peak_	156.4 ± 33.2	140.7 ± 37.2	−15.8 [5.4 − 26.2]	−10.1%**
		RTD_50 ms_	895.3 ± 186.9	675.9 ± 135.8	−219.4 [13.9 – 424.7]	−24.5%**
		RTD_100 ms_	953.3 ± 224.5	749.2 ± 136.9	−204.1 [−4.5 – 412.7]	−21.4%**

*Significantly different from fatigue for *p* < 0.05.

**Significantly different from fatigue for *p* < 0.01.

***Significantly different from fatigue for *p* < 0.001.

*All data are presented as mean ± SD.*

**TABLE 2:** Effect of fatigue on conventional and functional H/Q ratios and RTD H/Q ratio.

	Velocity	Pre-fatigue	Post-fatigue	Mean Difference [95% CI]	Change
Conventional H/Q Ratio	H_con90_/Q_con90_	0.63 ± 0.09	0.61 ± 0.11	−0.02 [−0.05 – 0.10]	−3.1%
	H_con180_/Q_con180_	0.73 ± 0.13	0.75 ± 0.15	0.01 [−0.10 – 0.07]	+2.1%
Functional H/Q Ratio	H_ecc30_/Q_con90_	0.78 ± 0.14	0.82 ± 0.14	0.04 [0.04 – 0.12]	+5.2%
	H_ecc30_/Q_con180_	1.03 ± 0.19	1.36 ± 0.33	0.33 [0.19 – 0.47]	+32.0%***
RTD_50 ms_ H/Q Ratio	90°.s^–1^	1.10 ± 0.44	0.71 ± 0.26	−0.39 [−0.53 – –0.25]	−35.5%**
	180°.s^–1^	1.07 ± 0.59	0.96 ± 0.44	−0.11 [0.15 – 0.38]	−10.3%
RTD_100 ms_ H/Q Ratio	90°.s^–1^	0.91 ± 0.18	0.85 ± 0.29	−0.06 [−0.08 – 0.22]	−6.6%
	180°.s^–1^	0.96 ± 0.35	0.82 ± 0.31	–0.14 [0.19 – 0.47]	−14.5%*

*Significantly different from fatigue for *p* < 0.05.

**Significantly different from fatigue for *p* < 0.01.

***Significantly different from fatigue for *p* < 0.001.

*All data are presented as mean ± SD.*

**FIGURE 3:**
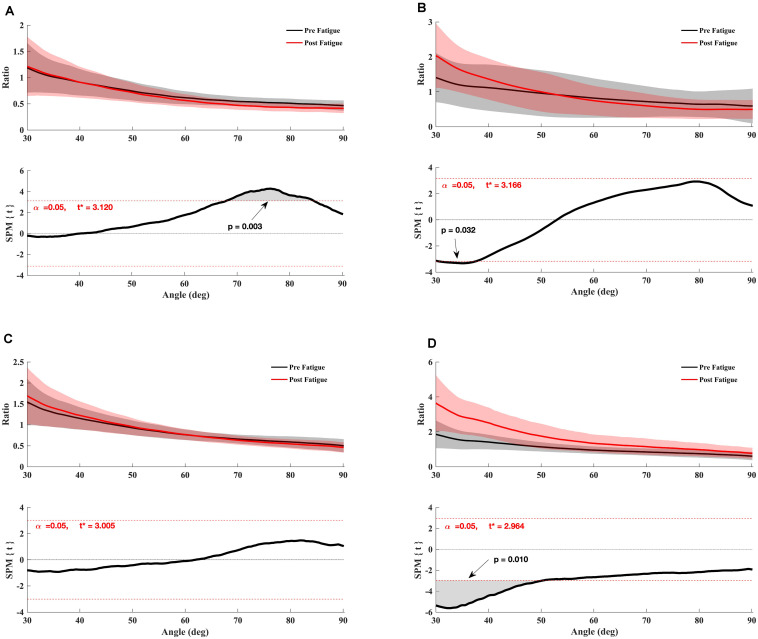
Effect of fatigue on the angle-specific ratio. **(A)** H/Q_conv90θ_, **(B)** H/Q_conv180θ_, **(C)** H/Q_func90θ_, **(D)** H/Q_func180θ_. Mean ± 95% confidence interval angle-series (upper panel) and corresponding SPM vector field statistical analysis (lower panel). The red dotted line represents the critical random field, the threshold value, *t* statistical set for α = 0.05. The *p* < 0.05 portion of the curve is shaded in gray.

Also, the paired *t*-test SPM analysis showed a significant increase in the conventional *H*_con180θ_/*Q*_con180θ_ ratio between 30° and 32° knee angle (*p* = 0.032) (Figure 3) and the functional *H*_ecc30θ_/*Q*_con180θ_ ratio between 30° and 48° of knee angle range of motion (*p* = 0.01) (Figure 3) after fatigue. The conventional ratio *H*_con90θ_/*Q*_con90θ_ decreased significantly after fatigue for the knee angle range of motion between 68° and 88° degrees (*p* = 0.003) (Figure 3). Nevertheless, no significant effect of fatigue was indicated for dynamic functional ratio *H*_ecc30θ_ /*Q*_con90θ_ (*p* > 0.05) (Figure 3).

### Rate of Torque Development and RTD H/Q Ratios

Absolute RTD_50 ms_ and RTD_100 ms_ reduced significantly after fatigue for both knee extensors and knee flexor at 90°.s^–1^, 180°.s^–1^, and −30°.s^–1^ (all *p* < 0.05) (Table 1). Meanwhile, Holm’s Bonferroni *post hoc* tests indicated that the reduction in RTD was higher at an angular velocity of 180°.s^–1^ (all *p* < 0.01). RTD_50 ms_ H/Q ratio at 90°.s^–1^ and RTD_50 ms_ at 180°.s^–1^ reduced following the fatigue protocol (all *p* < 0.05) (Table 2). In contrast, RTD_100 ms_ H/Q ratio at 90°.s^–1^ and RTD_50 ms_ at 180°.s^–1^ were not changed after the fatigue protocol (*p* > 0.05) (Table 2).

### Muscular Activity

The level of activation (RMS_mean_) during maximal contractions was not changed after the fatigue protocol, whatever the muscle and irrespective of the knee’s angular velocity (all *p* > 0.05) (Tables 3–5). In contrast, The RER_50 ms_, RER_100 ms_ were significantly reduced after the fatigue protocol for all the muscles (e.g., biceps femoris [BF], semitendinosus [ST], rectus femoris [RF], vastus medial [VM], vastus lateralis [VL]) (all *p* < 0.05), irrespective of angular velocity (i.e., 180°.s^–1^, 90°.s^–1^, −30°.s^–1^) (Tables 3–5).

**TABLE 3:** Effect of fatigue on mean RMS EMG (RMS_mean_) in% of maximal EMG signal, EMG rise (RER) in %.s^–1^ for hamstring (BF, biceps femoris; ST, semitendinosus) and quadriceps (RF, rectus femoris; VL, vastus lateralis; VM, vastus medialis) at 180°.s^–1^.

Velocity [°. s^–1^]	Muscle	EMG	Pre-fatigue	Post-fatigue	Mean Difference [95% CI]	Change [%]
180	BF	RER_50 ms_	334.7 ± 128.8	240.3 ± 93.1	−94.3 [27.8 − 161.0]	−28.2%**
		RER_100 ms_	311.0 ± 118.8	220.2 ± 72.9	−81.8 [20.1 − 143.4]	−26.4%**
		RMS_mean_	53.1 ± 11.7	59.7 ± 15.1	6.6 [4.1 − 17.1]	12.4%
	ST	RER_50 ms_	415.0 ± 189.1	277.1 ± 136.3	−137.9 [46.0 − 229.7]	−33.2%***
		RER_100 ms_	344.3 ± 120.9	247.1 ± 88.6	−97.1 [32.6 − 161.7]	−28.2%***
		RMS_mean_	56.6 ± 9.5	60.7 ± 10.6	4.1 [−1.7 − 6.5]	7.2%
	RF	RER_50 ms_	392.7 ± 220.3	254.2 ± 168.7	−138.5 [55.9 − 221.1]	−35.3%*
		RER_100 ms_	333.3 ± 137.4	273.9 ± 130.3	−59.4 [−3.7 − 122.6]	−17.8%*
		RMS_mean_	62.7 ± 4.6	63.7 ± 11.7	0.9 [−10.0 − 8.2]	1.4%
	VL	RER_50 ms_	493.3 ± 252.1	327.5 ± 145.3	165.9 [30.5 − 301.2]	−33.7%*
		RER_100 ms_	393.3 ± 141.9	308.6 ± 86.6	−84.8 [1.4 − 168.1]	−21.6%*
		RMS_mean_	73.9 ± 6.6	68.2 ± 8.9	−5.7 [0.8 − 10.5]	−7.7%
	VM	RER_50 ms_	383.9 ± 214.3	288.7 ± 175.2	−95.1 [−3.0 − 193.1]	−24.8%*
		RER_100 ms_	321.3 ± 147.5	263.1 ± 145.8	−58.2 [20.3 − 193.1]	−18.1%*
		RMS_mean_	55.1 ± 9.2	51.6 ± 9.7	3.4 [−1.6 − 8.5]	−6.2%

*Significantly different from fatigue for *p* < 0.05.

**Significantly different from fatigue for *p* < 0.01.

***Significantly different from fatigue for *p* < 0.001.

*All data are presented as mean ± SD.*

**TABLE 4:** Effect of fatigue on mean RMS EMG (RMS_mean_) in %, of maximal EMG signal EMG rise (RER) in %.s^–1^ for hamstring (BF, biceps femoris; ST, semitendinosus) and quadriceps (RF, rectus femoris; VL, vastus lateralis; VM, vastus medialis) at 90°.s^–1^.

Velocity [°. s^–1^]	Muscle	EMG	Pre-fatigue	Post-fatigue	Mean Difference [95% CI]	Change [%]
90	BF	RER_50 ms_	520.5 ± 181.4	344.9 ± 217.1	−175.6 [37.7 − 313.6]	−33.7%*
		RER_100 ms_	447.8 ± 158.3	333.3 ± 166.8	−114.5 [3.9 − 225.1]	−25.6%*
		RMS_mean_	66.1 ± 7.5	62.9 ± 8.3	−3.1 [−4.4 − 10.6]	−4.7%
	ST	RER_50 ms_	545.1 ± 236.5	384.7 ± 196.8	−160.4 [53.7 − 267.1]	−29.4%*
		RER_100 ms_	428.2 ± 148.2	343.1 ± 107.5	−85.1 [3.8 − 166.]	−19.9%*
		RMS_mean_	60.7 ± 8.1	64.6 ± 11.2	−3.8 [−7.1 − 17.9]	6.3%
	RF	RER_50 ms_	548.7 ± 310.2	344.9 ± 269.4	−203.7 [54.7 − 352.1]	−37.1%*
		RER_100 ms_	436.7 ± 192.1	334.5 ± 190.3	−102.1 [24.0 − 180.5]	−23.4%*
		RMS_mean_	68.3 ± 6.9	62.7 ± 14.0	−5.6 [2.8 − 13.9]	−8.2%
	VL	RER_50 ms_	533.4 ± 204.7	343.9 ± 188.9	−189.4 [55.5 − 323.4]	−35.5%***
		RER_100 ms_	442.1 ± 140.1	347.6 ± 140.5	−94.5 [26.6 − 162.3]	−21.4%*
		RMS_mean_	70.5 ± 6.9	71.7 ± 8.1	−1.1 [−5.3 − 3.1]	−1.6%
	VM	RER_50 ms_	538.7 ± 226.7	371.7 ± 154.9	−167.0 [59.4 − 274.4]	−31.0%**
		RER_100 ms_	427.8 ± 138.6	336.4 ± 140.4	−91.4 [17.6 − 165.1]	−21.4%*
		RMS_mean_	68.5 ± 7.2	72.3 ± 6.4	−3.8 [−8.9 − 1.3]	−5.5%

*Significantly different from fatigue for *p* < 0.05.

***Significantly different from fatigue for *p* < 0.01.

***Significantly different from fatigue for *p* < 0.001.

*All data are presented as mean ± SD.*

**TABLE 5:** Effect of fatigue on mean RMS EMG (RMS_mean_) in % of maximal EMG signal, EMG rise (RER) in %. s^–1^ for hamstring (BF, biceps femoris; ST, semitendinosus) and quadriceps (RF, rectus femoris; VL, vastus lateralis; VM, vastus medialis) at −30°.s^–1^.

Velocity [°. s^–1^]	Muscle	EMG	Pre-fatigue	Post-fatigue	Mean Difference [95% CI]	Change [%]
−30	BF	RER_50 ms_	328.7 ± 148.9	257.9 ± 146.9	−70.7 [−45.3 − 186.9]	−21.5%*
		RER_100 ms_	389.1 ± 190.6	265.5 ± 148.1	−123.6 [10.4 − 239.9]	−31.8%”
		RMS_mean_	54.6 ± 9.9	49.1 ± 10.7	−5.5 [0.5 − 10.4]	−10.1%
	ST	RER_50 ms_	371.1 ± 235.6	299.9 ± 184.4	−71.1 [−20.3 − 161.5]	−19.2%*
		RER_100 ms_	370.8 ± 239.8	265.4 ± 148.1	−97.8 [−7.8 − 203.4]	−26.4%**
		RMS_mean_	49.8 ± 11.1	48.4 ± 10.2	−1.3 [−7.5 − 10.2]	−2.6%

*Significantly different from fatigue for *p* < 0.05.

**Significantly different from fatigue for *p* < 0.01.

***Significantly different from fatigue for *p* < 0.001.

*All data are presented as mean ± SD.*

**FIGURE 4:**
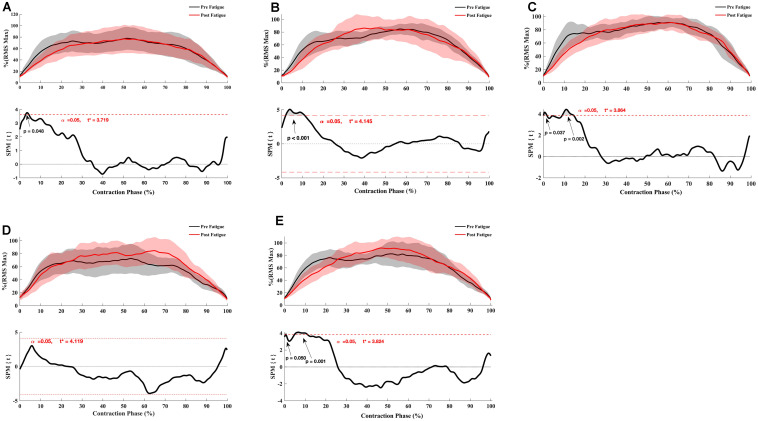
Effect of fatigue on EMG RMS curve of hamstring and quadriceps during knee flexion and extension at 180°.s^–1^. **(A)** Vastus Medialis, **(B)** Rectus Femoris, **(C)** Vastus Lateralis, **(D)** Biceps Femoris Long-Head, **(E)** Semitendinosus. Mean ± 95% confidence interval contraction period-series (upper panel) and corresponding SPM vector field statistical analysis (lower panel). The red dotted line represents the critical random field, the threshold value, *t* statistical set for α = 0.05. The *p* < 0.05 portion of the curve is shaded in gray.

**FIGURE 5:**
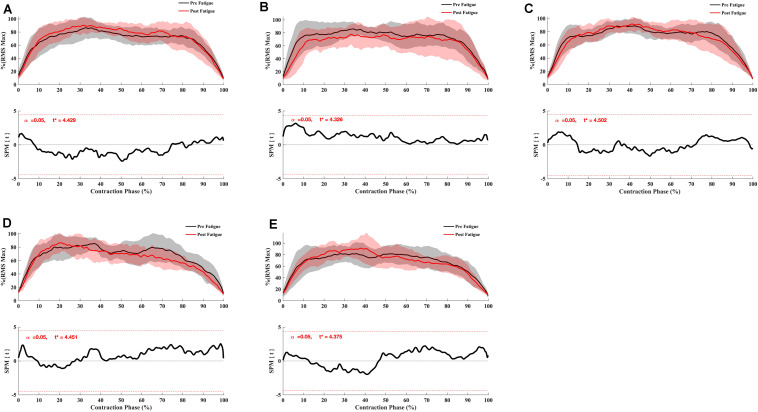
Effect of fatigue on EMG RMS curve of hamstring and quadriceps during knee flexion and extension at 90°.s^–1^. **(A)** Vastus Medialis, **(B)** Rectus Femoris, **(C)** Vastus Lateralis, **(D)** Biceps Femoris Long-Head, **(E)** Semitendinosus. Mean ± 95% confidence interval contraction period-series (upper panel) and corresponding SPM vector field statistical analysis (lower panel). The red dotted line represents the critical random field, the threshold value, *t* statistical set for α = 0.05. The *p* < 0.05 portion of the curve is shaded in gray.

**FIGURE 6:**
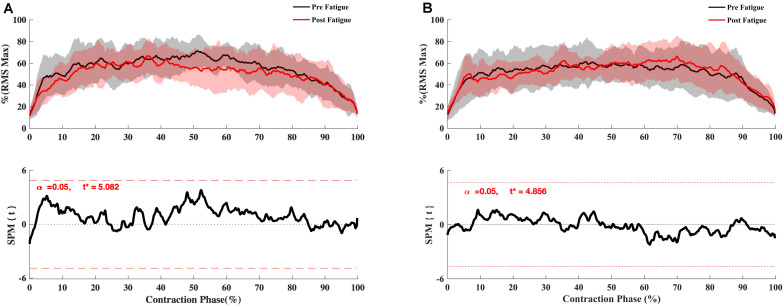
Effect of fatigue on EMG RMS curve of hamstring during eccentric knee extension at −30°.s^–1^. **(A)** Biceps Femoris Long-Head, **(B)** Semitendinosus. Mean ± 95% confidence interval contraction period-series (upper panel) and corresponding SPM vector field statistical analysis (lower panel). The red dotted line represents the critical random field, the threshold value, *t* statistical set for α = 0.05. The *p* < 0.05 portion of the curve is shaded in gray.

The SPM *t*-test results showed the muscular activation to be slightly slower at 180°.s^–1^ in the early phase of contraction for VM (*p* = 0.048), RF (*p* < 0.001), vastus lateralis (*p* = 0.037, *p* = 0.002), ST (*p* = 0.05, *p* = 0.001) but not for biceps femoris (*p* > 0.05) (Figure 4), whereas no significant effect of fatigue on the RMS curve was observed at 90°. s^–1^ (Figure 5) and −30°.s^–1^ (all *p* > 0.05) (Figure 6).

**FIGURE 7:**
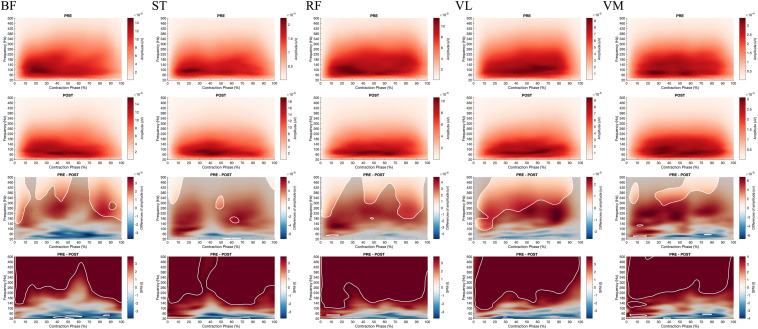
From top to bottom; **rows 1 and 2**: EMG Frequency-Time map during concentric contractions for BF, ST, RF, VL, VM before (PRE, **row 1**) and after (POST, **row 2**) the fatiguing exercise respectively; **row 3**: The magnitude of differences between PRE and POST. The enlighten clusters correspond to significant differences, considering the significance of the two-way ANOVA (*p* < 0.05 after Bonferonni correction); **row 4**: Results of the *post hoc* tests (SPM *t*-tests). The enlighten clusters correspond to significant differences without considering the ANOVA (*p* < 0.05 after Bonferonni correction). BF, Biceps femoris; ST, Semitendinosus; RF, Rectus femoris; VL, Vastus lateralis; VM, Vastus medialis.

**FIGURE 8:**
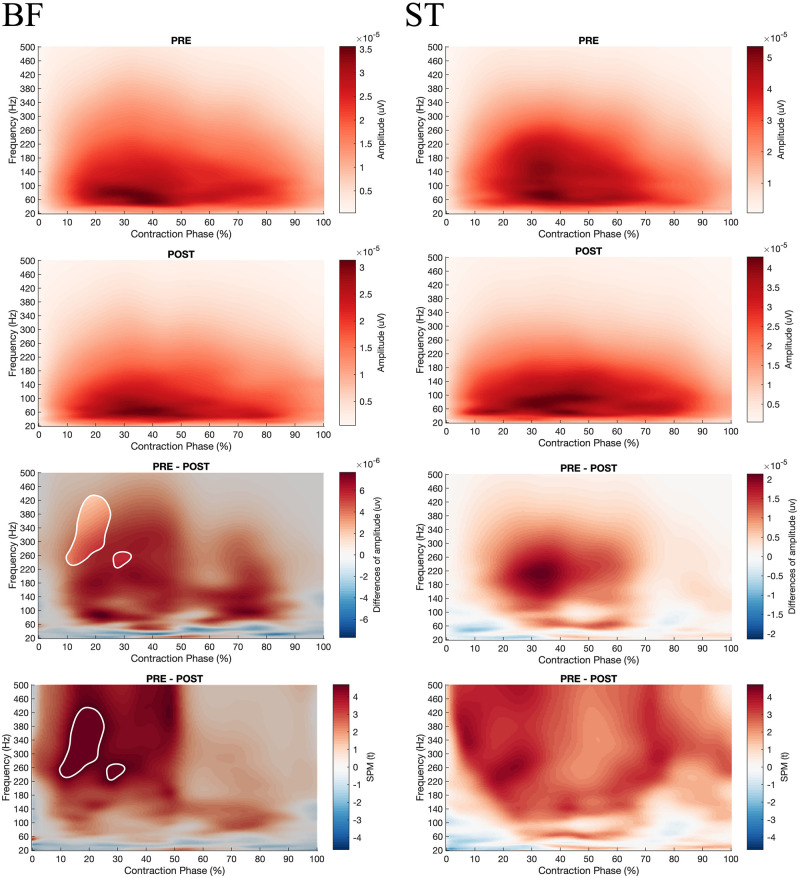
From top to bottom; **rows 1 and 2**: EMG Frequency-Time map during concentric contractions for BF and ST before (PRE, **row 1**) and after (POST, **row 2**) the fatiguing exercise respectively; **row 3**: The magnitude of differences between PRE and POST. The enlighten clusters correspond to significant differences (*p* < 0.05 after Bonferonni correction); **row 4**: Results of the *post hoc* tests (SPM *t*-tests). The enlighten clusters correspond to significant differences (*p* < 0.05 after Bonferonni correction). BF, Biceps femoris; ST, Semitendinosus.

Besides, the SPM ANOVA analysis on the EMG Frequency-Time maps indicated significant effect of fatigue (Pre vs. Post) (Figure 7) for all the muscles, whereas no effect of velocity (90°.s^–1^ vs. 180°.s^–1^) (Supplementary Figures 1–6). Similarly for all the muscles during concentric contractions, fatigue decreased the amplitude in high frequencies of EMG signals (above 100 Hz), while increasing the low frequencies amplitude (under 50 Hz) after 20% of contraction time. At the start of the contraction (0–20%), both high and low frequencies were decreased. Also, the SPM *t*-test revealed a significant fatigue effect on BF during the eccentric contraction (−30°.s^–1^), reducing the higher frequency component after fatigue (Figure 8), while no significant effect of fatigue was denoted for ST.

## Discussion

The goal of the present study was to analyze the H/Q ratio taking into account timing and angle specificity during a fresh and fatigued state. The major finding of this study demonstrated an equivalent decrease in force-generating capacity of knee flexors and extensors after fatigue. Moreover, fatigue modified angle-specific H/Q ratios and lowered the RTD and the EMG rise.

### Influence of Fatigue on Torque Production and H/Q Ratios

Overall, the present results indicated that there was an equivalent and marked reduction of the peak torque of knee flexor and extensor muscles at each velocity of contraction. This is not in line with previous studies, since Sangnier and Tourny reported a greater torque loss in hamstring (70 ± 8.05%) compared to quadriceps (60 ± 9.50%) muscles after 25 maximal knee flexions/extensions at 180°.s^–1^ (Sangnier and Tourny-Chollet, 2007). However, Kawabata et al. (2000) found that the strength decreased index (SDI) was more substantial for the quadriceps than for hamstring muscles in soccer players (Kawabata et al., 2000). Since one study reported a high proportion of fast-twitch fibers in the hamstring muscles in soccer players (Garrett et al., 1984), a higher fatigability of knee flexors was expected. However, the recent study by Evangelidis et al. (2017) reported a balanced myosin heavy chain composition of the biceps femoris long head (BFlh) comparable to the vastus muscle (Evangelidis et al., 2017). Nevertheless, the analysis of conventional and functional H/Q ratios demonstrated that fatigue did not negatively impact muscle balance. Surprisingly, the functional *H*_ecc30_/*Q*_con180_ ratio was even improved after fatigue. This could be explained by the fact that the fatigue session induced a deeper strength loss during the isokinetic contractions performed at 180°.s^–1^ compared to other velocities. Whatever the origin of these modifications, it seems that the increased injury risk of HSIs after fatigue or at the end of matches may not be explained by an impaired functional and conventional H/Q ratio. However, it has been demonstrated that the impact of fatigue on H/Q ratios can be advantageously reinforced using SPM analysis on torque-angle curves (Alhammoud et al., 2019). Surprisingly, the SPM analysis showed that H/Q ratios were improved after fatigue at low knee angles in a relatively extended knee position. In conclusion, SPM analysis demonstrated that angle-specific H/Q ratio modifications after fatigue could not explain the increased occurrence of the HSIs and ACL injury at the end of an exercise.

### Influence of Fatigue on Rapid Torque Production

Another factor that is known to be sensitive to fatigue and related to injury risk is the ability to produce force rapidly, which can be explored through RTD (Grazioli et al., 2019). It is interesting to observe that peak torque reduction was stronger at 180°.sec^–1^ compared to other isokinetic velocities. This point may be explained by the slowing of cross-bridge cycling rate after fatigue (De Ruiter and De Haan, 2001) and by the higher fatigability of fast-twitch fibers (Hautier et al., 1996). Similarly, an increased decline in explosive capacity RTD in the early phase of maximal contraction was observed after fatigue. This reduction in explosive capacity seems to be more marked than the peak torque decrease in line with Morel et al. (2015). This can be explained by alterations of neural and contractile factors (Andersen and Aagaard, 2006; Maffiuletti et al., 2016; Rodriguez-Rosell et al., 2018). Additionally, a significant reduction in the RTD_50 ms_ H/Q ratios was observed after fatigue, indicating that the ability to strengthen and stabilize the knee during the first milliseconds of a contraction was significantly altered after fatigue (Grazioli et al., 2019). This is particularly interesting considering the study by Correia et al. (2020), which reported a slight trend for a lower RTD H/Q ratio at 50 ms in previously injured hamstrings (Correia et al., 2020), and the results of Grazioli et al. (2019), which revealed that the RTD H/Q ratio in the early phase of contraction decreased by ∼24% after match-induced fatigue (Grazioli et al., 2019). Considering that the ability to produce torque during the first milliseconds of a contraction is considered as a critical factor for performance (Aagaard et al., 2002) and a determinant of injury risk (Grazioli et al., 2019; Correia et al., 2020), it can be hypothesized that the increased risk of ACL injury after fatigue could be partly explained by the decrease in rapid force capacities RTD and RTD H/Q ratios rather than conventional or functional maximal H/Q ratio modifications.

### Influence of EMG on Rapid Torque Capacities After Fatigue

The modifications of H/Q ratios and RTD after fatigue may be explained by alterations of neural and contractile factors (Morel et al., 2015; Rodriguez-Rosell et al., 2018). The present protocol did not enable us to explore the contractile factors, but the analysis of the EMG signal may provide insights about the causes of torque losses after fatigue. Firstly, the decrease in maximal torque measured within the fatigue protocol and during the maximum contractions was not associated with a reduction in the average level of RMS_mean_ EMG (Tables 3–5). It can be considered that the average value of EMG RMS correctly described muscle activation during maximal and submaximal efforts which suggests that the capacity of the central nervous system to activate muscles entirely was unchanged. This is partly consistent with Morel et al. (2015), who reported a small and non-significant decrease in the level of voluntary activation (−4.3 ± 1.6%, *p* = 0.08) after repeated maximal knee extension to 240°.s^–1^ (Morel et al., 2015). However, if similar levels of maximum activation can be reached when the contraction time is long enough, it has been shown that this does not reflect the ability of the central nervous system to maximally activate the muscle during the first 100 ms of the contraction (Morel et al., 2015). The present results show a significant decrease in RER for knee extensors and flexors regardless of the contraction velocity. Of course, RTD is influenced by many factors such as tissue stiffness, muscle typology, cross-bridge kinetics, and neural drive (Edman and Josephson, 2007). However, in the present study, the decrease in RER could be related to the reduction in RTD (Morel et al., 2015). These results suggest that the ability of the central nervous system to activate the muscle quickly and to the maximum at the start of the contraction was altered after fatigue. Considering the study by Morel et al. (2015), this point should be analyzed considering the decrease in frequency content of the EMG signal (Morel et al., 2015). Present results show that repeated maximal contractions can induce alterations of the recruitment and firing frequency of the motor units during the early part of the contraction (Aagaard et al., 2002), which result in lowering RER and RTD. In the present study a SPM analysis was performed on the EMG Frequency-Time maps and the results demonstrated a significant reduction of the power contained in the high frequencies of the EMG signal for all the studied muscles during the concentric contractions, and merely for biceps femoris during eccentric contraction, respectively. However, this reduction was not specifically observed in the first 100 ms of the contractions as reported by Morel et al. (2015) but appeared all over the contractions except for the eccentric contractions of the BF muscle which demonstrated a significant reduction of the EMG frequency specifically during the first 30% of the movement. Thus, considering the study by Zebis et al. (2009), such a difference in rapid muscle activation in hamstring and quadriceps muscles may be predictive of future ACL injury (Zebis et al., 2009). It seems that fatigue can, therefore, increase the risk of injury in soccer players by decreasing their ability to activate muscles during the first 100 ms of a contraction which may alter the knee joint stability.

### Limitation

The study has some limitations that should be kept in mind. Firstly, the present study’s sample size is relatively small and not having professional players within the sample may be considered as a limitation. Secondly, no electrical or magnetic stimulation was used in the present study and if the analysis of EMG signal can give some insights about the muscular activation during the exercise it didn’t permit to conclude about the origin of fatigue. Lastly, it should be kept in mind that the RTD has been calculated during dynamic isokinetic contractions and it is well known that the isokinetic dynamometers are often more compliant at the beginning of the contraction and noisier than strain gauges which may induce a lower measurement precision for RTD calculation (Maffiuletti et al., 2016). Furthermore, it is impossible at the present time to determine precisely the predictive value of the studied variables in terms of injuries. However, we believe that this paper propose new and convergent insights that may be used in future studies to determine this predictive value.

## Conclusion

The purpose of this study was to assess the effect of fatigue on angle-specific H/Q ratios, RTD H/Q ratios, and muscle activation in soccer players in order to better understand the augmented risk of HSIs injury at the end of a match. This study demonstrates that conventional and functional ratio were not decreased after fatigue and nor was the ability to maximally activate the muscles. However, the rapid hamstring to quadriceps ratio RTD H/Q were more affected in association with an overall slow down of RER during the first 100 ms of the contraction and a decrease in frequency content of the EMG signal. Besides, fatigue seems to reduce the potential for knee joint stability due to the RER deficit during the initial phase of contraction. In accordance with recent studies, present outcomes may confirm the importance of explosive force testing before and after fatigue to improve performance and prevent injuries including (i.e., HSIs, ACL injury). In summary, the results of this study show that functional and conventional H/Q ratios carried out during the pre-season tests are not sensitive to fatigue. It seems, in agreement with many authors, that the evaluation of the capacities of rapid force production during the first 100 ms of a contraction, with and without fatigue, is more appropriate to evaluate and prepare soccer players.

### Practical Recommendation

This study indicated that the traditional H/Q ratios calculated using the peak torque were not sensitive to fatigue whereas dynamic angle specific ratios seemed to give additional information about muscle weakness at some knee angles which may justify the utilization of this kind of analysis in soccer players evaluation before and after fatigue. Moreover, it appeared that RTD H/Q ratios were modified after fatigue. Considering that a short period (i.e., less than 50 ms) is frequently available to stabilize the knee joint through knee muscle contraction after ground contact moment during braking or dropping maneuver 3, 4 one may consider that the measurement of this parameter with and without fatigue is relevant for injury prevention in soccer. From a general point of view and as hypothesized by other authors, it seems that the evaluation of knee stability in soccer players may now incorporate specific angle H/Q ratios, rapid torque development capacities of extensor and flexor muscles and be performed under fatigue state to provide efficient and meaningful indicators to players, coaches, and therapists.

## Data Availability Statement

The raw data supporting the conclusions of this article will be made available by the authors, without undue reservation.

## Ethics Statement

The studies involving human participants were reviewed and approved by “Sud-Est II” of Lyon. The patients/participants provided their written informed consent to participate in this study.

## Author Contributions

QZ, BM, and CH conceived and designed the experiments, and wrote the manuscript. QZ and CH performed the experiments. QZ, RT, and CH analyzed the data and contributed materials and analysis tools. All the authors contributed to the article and approved the submitted version.

## Conflict of Interest

The authors declare that the research was conducted in the absence of any commercial or financial relationships that could be construed as a potential conflict of interest.

## Supplementary Material

Supplementary Figure 1 Two-way ANOVA SPM (Fatigue ^∗^ Velocity) EMG Frequency-Time maps analysis for BF, ST, RF, VL, and VM. From left to right; **column 1**: the magnitude of differences from interaction Fatigue ^∗^ Velocity, **column 2**: the magnitude of differences from Fatigue (*p* < 0.05), **column 3**: the magnitude of differences from Velocity; the enlighten clusters correspond to significant differences of the two-way ANOVA (*p* < 0.05). BF, Biceps femoris; ST, Semitendinosus; RF, Rectus femoris; VL, Vastus lateralis; VM, Vastus medialis.

Supplementary Figure 2 From top to bottom; **rows 1 and 2**: EMG Frequency-Time map during concentric contractions for Biceps Femoris before (PRE, **row 1**) and after (POST, **row 2**) the fatiguing exercise with/without the interaction angular velocity (90°.s^–1^, 180°.s^–1^)respectively; **row 3**: the magnitude of differences between PRE and POST. The enlighten clusters correspond to significant differences, considering the significance of the two-way ANOVA (*p* < 0.05 after Bonferonni correction); **row 4**: results of the *post hoc* tests (SPM *t*-tests). The enlighten clusters correspond to significant differences without considering the ANOVA (*p* < 0.05 after Bonferonni correction).

Supplementary Figure 3 From top to bottom; **rows 1 and 2**: EMG Frequency-Time map during concentric contractions for Semitendinosus before (PRE, **row 1**) and after (POST, **row 2**) the fatiguing exercise with/without the interaction angular velocity (90°.s^–1^, 180°.s^–1^) respectively; **row 3**: the magnitude of differences between PRE and POST. The enlighten clusters correspond to significant differences, considering the significance of the two-way ANOVA (*p* < 0.05 after Bonferonni correction); **row 4**: results of the *post hoc* tests (SPM *t-*tests). The enlighten clusters correspond to significant differences without considering the ANOVA (*p* < 0.05 after Bonferonni correction).

Supplementary Figure 4 From top to bottom; **rows 1 and 2**: EMG Frequency-Time map during concentric contractions for Rectus Femoris before (PRE, **row 1**) and after (POST, **row 2**) the fatiguing exercise with/without the interaction angular velocity (90°.s^–1^, 180°.s^–1^) respectively; **row 3**: The magnitude of differences between PRE and POST. The enlighten clusters correspond to significant differences, considering the significance of the two-way ANOVA (*p* < 0.05 after Bonferonni correction); **row 4**: results of the *post hoc* tests (SPM *t*-tests). The enlighten clusters correspond to significant differences without considering the ANOVA (*p* < 0.05 after Bonferonni correction).

Supplementary Figure 5 From top to bottom; **rows 1 and 2**: EMG Frequency-Time map during concentric contractions for Vastus Lateralis before (PRE, **row 1**) and after (POST, **row 2**) the fatiguing exercise with/without the interaction angular velocity (90°.s^–1^, 180°.s^–1^) respectively; **row 3**: The magnitude of differences between PRE and POST. The enlighten clusters correspond to significant differences, considering the significance of the two-way ANOVA (*p* < 0.05 after Bonferonni correction); **row 4**: results of the *post hoc* tests (SPM *t*-tests). The enlighten clusters correspond to significant differences without considering the ANOVA (*p* < 0.05 after Bonferonni correction).

Supplementary Figure 6 From top to bottom; **rows 1 and 2**: EMG Frequency-Time map during concentric contractions for Vastus Medialis before (PRE, **row 1**) and after (POST, **row 2**) the fatiguing exercise with/without the interaction angular velocity (90°.s^–1^, 180°.s^–1^) respectively; **row 3**: The magnitude of differences between PRE and POST. The enlighten clusters correspond to significant differences, considering the significance of the two-way ANOVA (*p* < 0.05 after Bonferonni correction); **row 4**: Results of the *post hoc* tests (SPM *t*-tests). The enlighten clusters correspond to significant differences without considering the ANOVA (*p* < 0.05 after Bonferonni correction).
