# Supplementary figures and images for: Influence of Fatigue on the Rapid Hamstring/Quadriceps Force Capacity in Soccer Players
Source: Front Physiol. 2021 Feb 5;12:627674. doi: 10.3389/fphys.2021.627674 (PMC7893113; doi:10.3389/fphys.2021.627674)

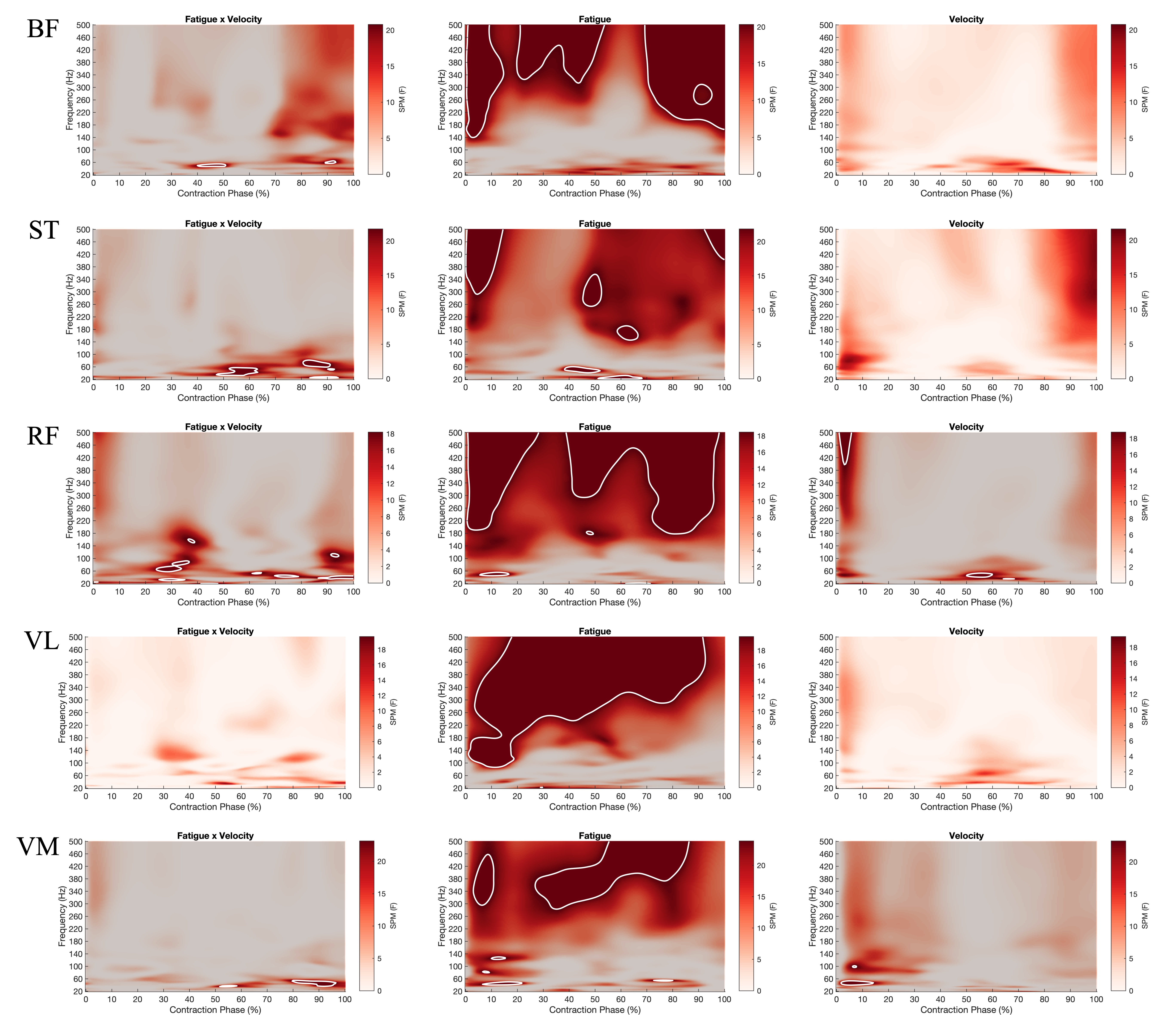

Supplement: Supplementary Figure 1 — Two-way ANOVA SPM (Fatigue ∗ Velocity) EMG Frequency-Time maps analysis for BF, ST, RF, VL, and VM. From left to right; column 1: the magnitude of differences from interaction Fatigue ∗ Velocity, column 2: the magnitude of differences from Fatigue (p < 0.05), column 3: the magnitude of differences from Velocity; the enlighten clusters correspond to significant differences of the two-way ANOVA (p < 0.05). BF, Biceps femoris; ST, Semitendinosus; RF, Rectus femoris; VL, Vastus lateralis; VM, Vastus medialis. [file Image_1.jpeg]

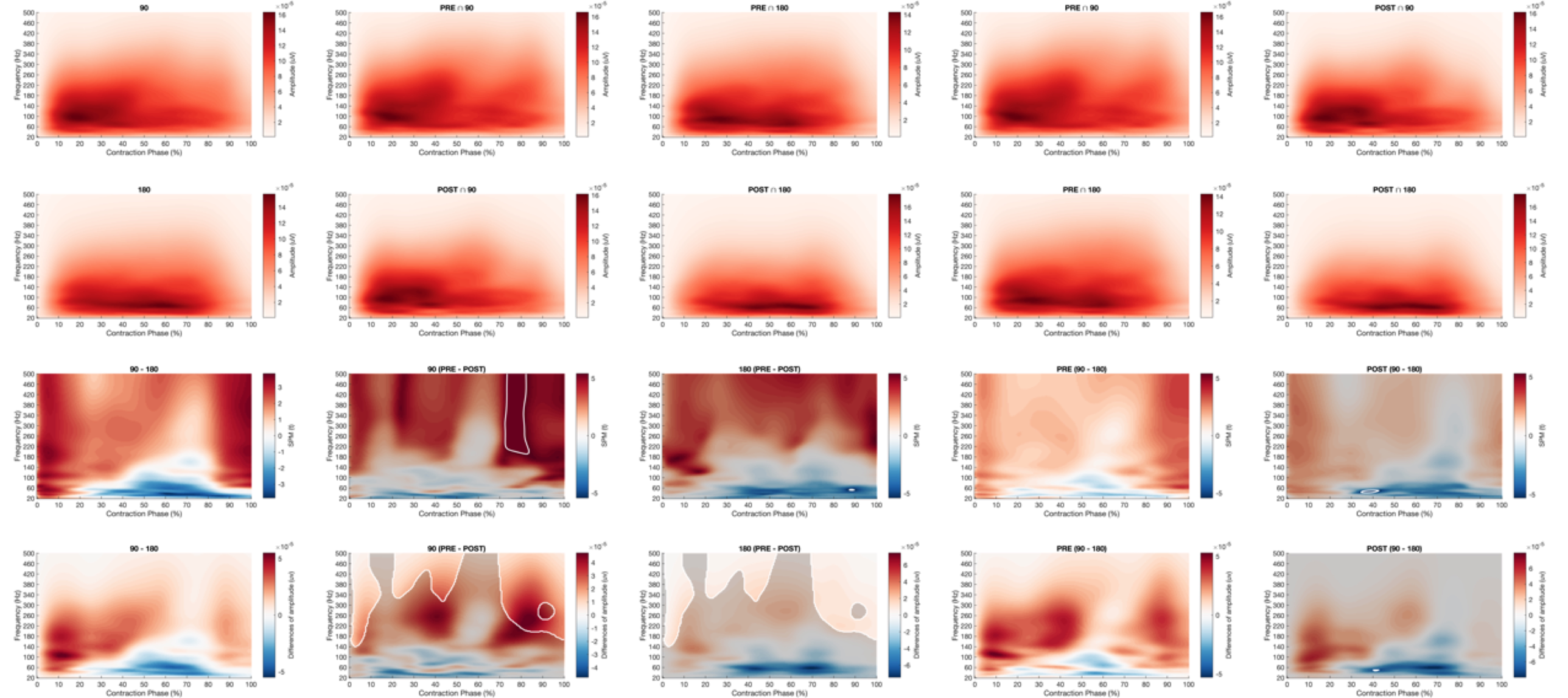

Supplement: Supplementary Figure 2 — From top to bottom; rows 1 and 2: EMG Frequency-Time map during concentric contractions for Biceps Femoris before (PRE, row 1) and after (POST, row 2) the fatiguing exercise with/without the interaction angular velocity (90°.s–1, 180°.s–1)respectively; row 3: the magnitude of differences between PRE and POST. The enlighten clusters correspond to significant differences, considering the significance of the two-way ANOVA (p < 0.05 after Bonferonni correction); row 4: results of the post hoc tests (SPM t-tests). The enlighten clusters correspond to significant differences without considering the ANOVA (p < 0.05 after Bonferonni correction). [file Image_2.jpeg]

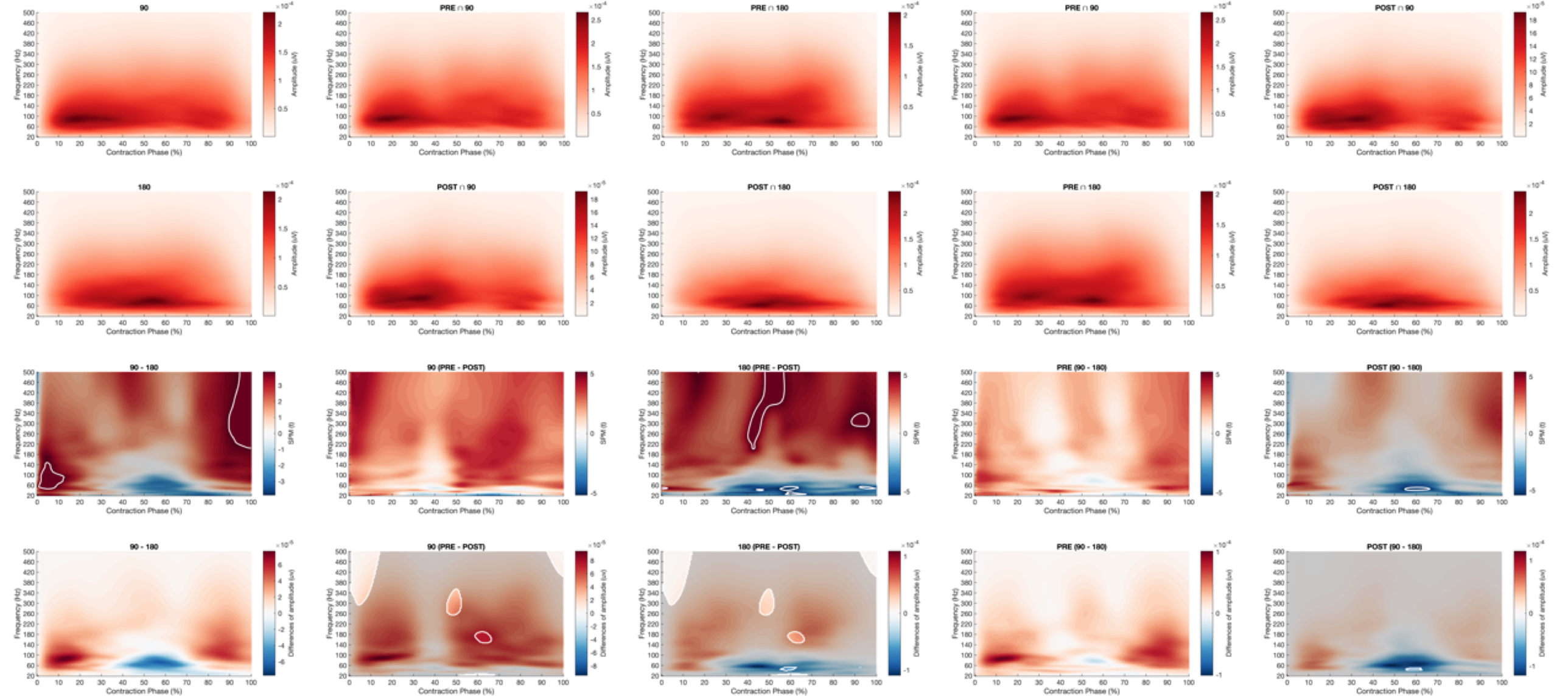

Supplement: Supplementary Figure 3 — From top to bottom; rows 1 and 2: EMG Frequency-Time map during concentric contractions for Semitendinosus before (PRE, row 1) and after (POST, row 2) the fatiguing exercise with/without the interaction angular velocity (90°.s–1, 180°.s–1) respectively; row 3: the magnitude of differences between PRE and POST. The enlighten clusters correspond to significant differences, considering the significance of the two-way ANOVA (p < 0.05 after Bonferonni correction); row 4: results of the post hoc tests (SPM t-tests). The enlighten clusters correspond to significant differences without considering the ANOVA (p < 0.05 after Bonferonni correction). [file Image_3.jpeg]

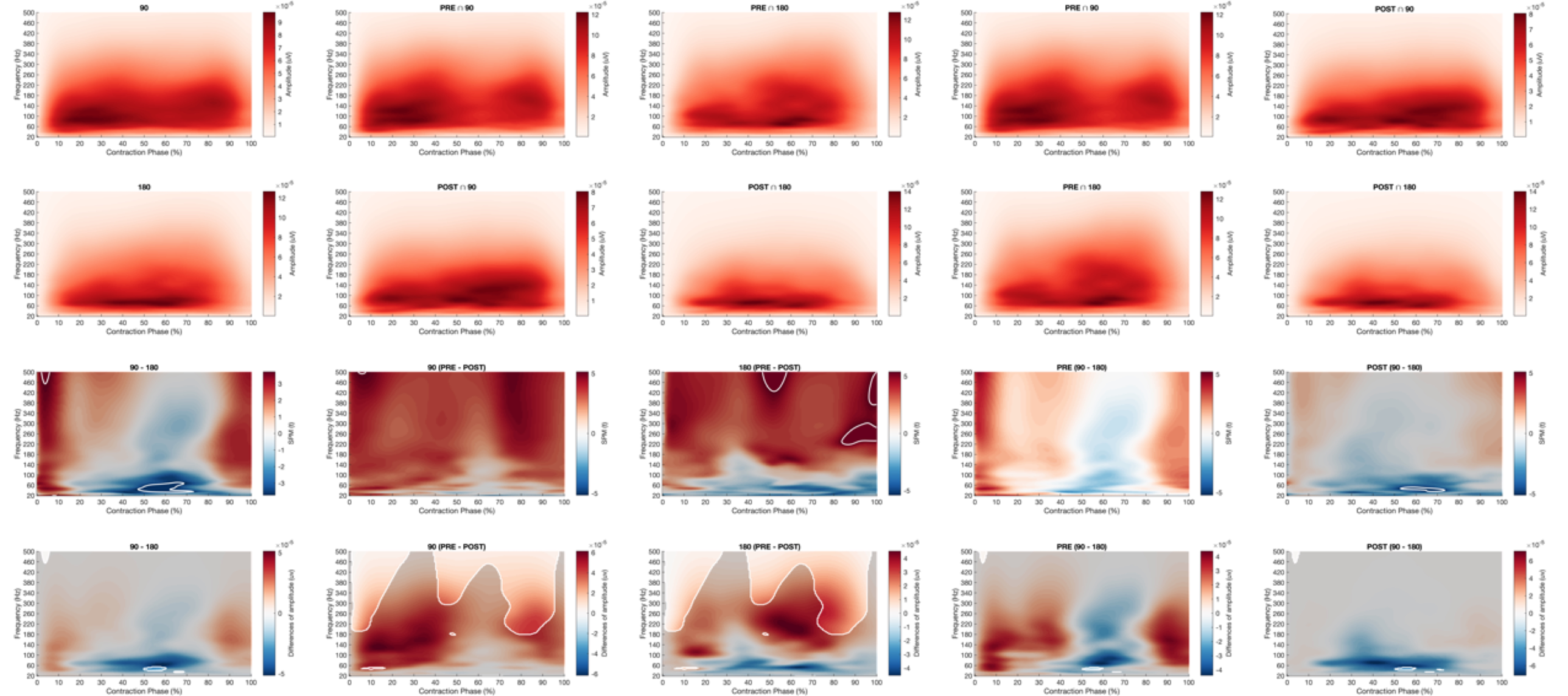

Supplement: Supplementary Figure 4 — From top to bottom; rows 1 and 2: EMG Frequency-Time map during concentric contractions for Rectus Femoris before (PRE, row 1) and after (POST, row 2) the fatiguing exercise with/without the interaction angular velocity (90°.s–1, 180°.s–1) respectively; row 3: The magnitude of differences between PRE and POST. The enlighten clusters correspond to significant differences, considering the significance of the two-way ANOVA (p < 0.05 after Bonferonni correction); row 4: results of the post hoc tests (SPM t-tests). The enlighten clusters correspond to significant differences without considering the ANOVA (p < 0.05 after Bonferonni correction). [file Image_4.jpeg]

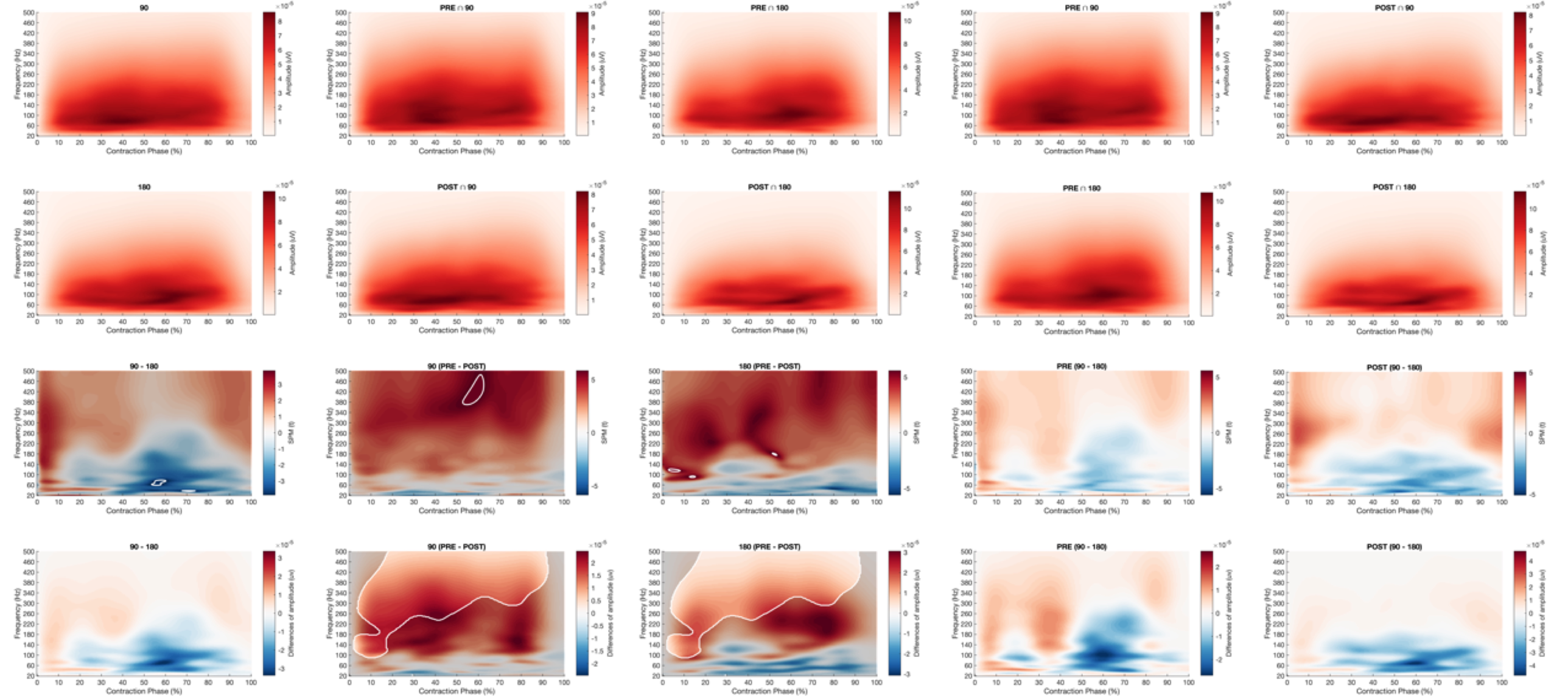

Supplement: Supplementary Figure 5 — From top to bottom; rows 1 and 2: EMG Frequency-Time map during concentric contractions for Vastus Lateralis before (PRE, row 1) and after (POST, row 2) the fatiguing exercise with/without the interaction angular velocity (90°.s–1, 180°.s–1) respectively; row 3: The magnitude of differences between PRE and POST. The enlighten clusters correspond to significant differences, considering the significance of the two-way ANOVA (p < 0.05 after Bonferonni correction); row 4: results of the post hoc tests (SPM t-tests). The enlighten clusters correspond to significant differences without considering the ANOVA (p < 0.05 after Bonferonni correction). [file Image_5.jpeg]

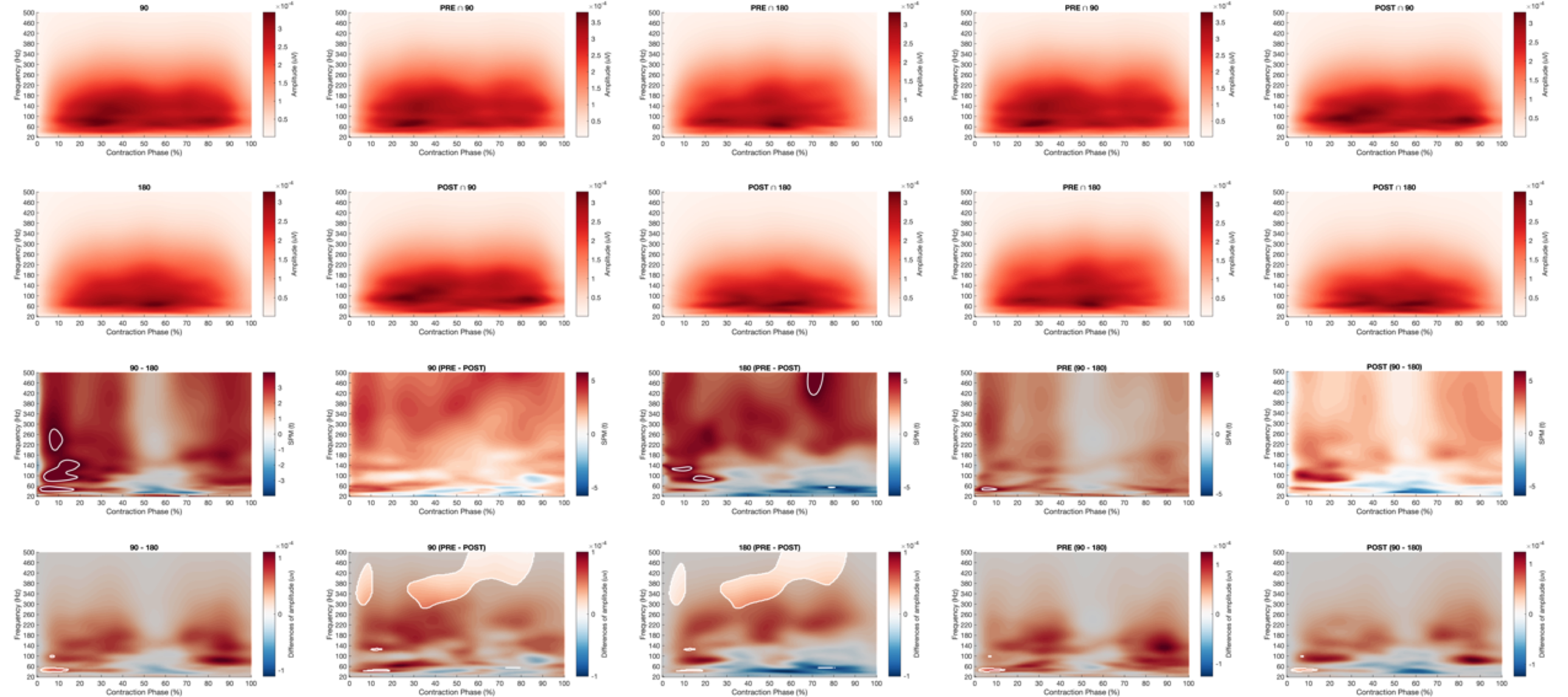

Supplement: Supplementary Figure 6 — From top to bottom; rows 1 and 2: EMG Frequency-Time map during concentric contractions for Vastus Medialis before (PRE, row 1) and after (POST, row 2) the fatiguing exercise with/without the interaction angular velocity (90°.s–1, 180°.s–1) respectively; row 3: The magnitude of differences between PRE and POST. The enlighten clusters correspond to significant differences, considering the significance of the two-way ANOVA (p < 0.05 after Bonferonni correction); row 4: Results of the post hoc tests (SPM t-tests). The enlighten clusters correspond to significant differences without considering the ANOVA (p < 0.05 after Bonferonni correction). [file Image_6.jpeg]
